# Glutamatergic Dysfunction and Synaptic Ultrastructural Alterations in Schizophrenia and Autism Spectrum Disorder: Evidence from Human and Rodent Studies

**DOI:** 10.3390/ijms22010059

**Published:** 2020-12-23

**Authors:** Ahmed Eltokhi, Andrea Santuy, Angel Merchan-Perez, Rolf Sprengel

**Affiliations:** 1 Department of Neurology and Epileptology, Hertie Institute for Clinical Brain Research, Tübingen University, D-72076 Tübingen, Germany; and.santuy@gmail.com; 2 Laboratorio Cajal de Circuitos Corticales, Centro de Tecnología Biomédica, Universidad Politécnica de Madrid, 28223 Madrid, Spain; amerchan@fi.upm.es; 3 Departamento de Arquitectura y Tecnología de Sistemas Informáticos, Universidad Politécnica de Madrid, 28223 Madrid, Spain; 4 Research Group of the Max Planck Institute for Medical Research at the Institute for Anatomy and Cell Biology, Heidelberg University, D-69120 Heidelberg, Germany

**Keywords:** glutamatergic system, synaptic ultrastructure, schizophrenia, autism spectrum disorder

## Abstract

The correlation between dysfunction in the glutamatergic system and neuropsychiatric disorders, including schizophrenia and autism spectrum disorder, is undisputed. Both disorders are associated with molecular and ultrastructural alterations that affect synaptic plasticity and thus the molecular and physiological basis of learning and memory. Altered synaptic plasticity, accompanied by changes in protein synthesis and trafficking of postsynaptic proteins, as well as structural modifications of excitatory synapses, are critically involved in the postnatal development of the mammalian nervous system. In this review, we summarize glutamatergic alterations and ultrastructural changes in synapses in schizophrenia and autism spectrum disorder of genetic or drug-related origin, and briefly comment on the possible reversibility of these neuropsychiatric disorders in the light of findings in regular synaptic physiology.

## 1. The Glutamatergic System

Excitatory neurotransmission in the brain is primarily glutamatergic; glutamatergic neurons in the non-stimulated cerebral cortex consume up to 80% of the total brain metabolic activity [1,2,3]. For the fast glutamatergic transmission, ionotropic glutamate-gated channels (iGluRs) are recruited. iGluRs are tetrameric, and each of the four subunits has four distinct structural protein domains: an extracellular N-terminal domain, an extracellular ligand-binding domain, a transmembrane channel, and an intracellular C-terminal domain [4]. In total, multiple subunits encoded by 18 different genes can contribute to the formation of iGluRs. Most of these subunits derive from different mRNA splice or pre-mRNA edited variants of genes coding for iGluR subunits, which increase the complexity of the ionotropic glutamatergic neurotransmission system. Based on their main agonist, iGluRs are divided into α-amino-3-hydroxy-5-methylisoxazole-4-propionate receptors (AMPARs), *N*-methyl-d-aspartate receptors (NMDARs), and kainate receptors (KARs). All members of these three receptor families are hetero- or homo-tetramers that are permeable to cations. With the exception of KARs that are only permeable to Na^+^, other glutamate-gated ion channels are also permeable to Ca^2+^. Thus, in response to glutamate activation, they increase the intracellular Ca^2+^ levels, thereby activating Ca^2+^-dependent intracellular responses upon glutamate stimulation. In the AMPAR family, the Na^+^ and Ca^2+^ permeability is usually strictly determined by their subunit composition; only AMPARs that lack the GluA2 subunit are permeable to Ca^2+^ (CP+AMPARs). CP+AMPARs can contribute to some forms of synaptic plasticity. For the induction of activity-induced synaptic transmission, CP+AMPARs are translocated from extra-postsynaptic to postsynaptic sites in response to intense presynaptic glutamate stimulation. Subsequently, activity-induced incorporated (CP+AMPARs) are replaced by Ca^2+^-impermeable AMPARs (CP-AMPARs) [5]. In NMDARs, the Ca^2+^ influx is blocked during regular glutamatergic signal transmission at resting membrane potential by a Mg^2+^ ion but is possible after repetitive stimulations that largely depolarize the synaptic membrane and displace the Mg^2+^ ion [6].

The interplay of synaptic iGluR subtypes is thus specialized to regulate the activity-dependent Ca^2+^ influx into the postsynapse, which can lead to either a transient or a long-term alteration of the synaptic efficacy. Glutamate sensing in the synaptic cleft is modified further by the presence of the G-protein-coupled metabotropic glutamate receptors (mGluRs), for slower responses to increased glutamate levels in the synaptic cleft. The mGluRs are classified into three groups based on the receptor structure and ligand sensitivity [7]. Group I (mGluR 1 and 5) is mainly postsynaptic, while groups II (mGluR 2 and 3) and III (mGluR 4, 6, 7, and 8) are primarily presynaptic and modulate the neurotransmitter release [2,8]. Group I works through the activation of phospholipase C, while group II and III mainly operate by decreasing cyclic AMP levels [8]. Their functions are also different: group I potentiates presynaptic glutamate release and postsynaptic NMDAR currents, and groups II and III inhibit presynaptic glutamate release [7]. 

This molecular complexity of the glutamate sensing system suggests that the glutamate receptors play a major role in the experience-dependent modulation of the CNS. This view was supported by the dependence of the expression of iGluRs and mGluRs on development. Thus, for example, the NMDAR subunit (GluN2A) reaches its adult expression level at postnatal day 12 (P12), while GluN2B mRNA levels fall after P12 in rats [9]; and in young mice, the hippocampal long-term potentiation (LTP), induced by 1 × 100 Hz tetanic stimulation of CA3 to CA1 synapses, is in part independent of the AMPAR GluA1 subunit [10], which is required for the induction of this LTP form in adult mice [11]. Similarly, the structural organization and numbers of synapses are modified postnatally, demonstrating that the maturation of synaptic transmission and plasticity is accompanied by a structural reorganization of synapses (see below). In this process, glutamatergic postsynaptic scaffolding proteins such as the SH3 and multiple ankyrin domain proteins (SHANKs) appear to play a central role. Similar to the complex glutamatergic receptor system, in mammals, the SHANKs form a huge family of scaffolding proteins encoded by three genes (*SHANK1*, *2*, and *3*) that can express multiple isoforms. 

The glutamatergic system plays a central role in neurotransmission and synaptic plasticity and its modulation during development, and it is not surprising that over the past 25 years, substantial pharmacological, genetic, and experimental evidence using genetically modified mice has highlighted the importance of glutamate not only in learning and memory but also in neuropsychiatric disorders such as schizophrenia (SZ). In particular, the numerous iGluR and SHANK genes and their isoforms suggested that the sensitivity to fast signaling of extracellular glutamate is central in neurodevelopment and learning. Mutations introduced by gene targeting verified this hypothesis and provided strong experimental evidence that gene mutations in *Gria1* and the NMDAR subunit 1, *Grin1*, are associated with SZ phenotypes. Mutations that disturb this glutamate-gated NMDAR and AMPAR Ca^2+^ signaling ultimately lead to a dysfunction of neuron-to-neuron communication, neuronal network dynamics, and thus responses to environmental stimuli, as detailed below.

## 2. The Glutamatergic System in Neuropsychiatric Disorders

### 2.1. Schizophrenia (SZ)

In humans, SZ is a complex mental disorder characterized by a combination of symptoms including delusions, hallucinations, disorganized speech or behavior, lack of motivation, and cognitive deficits with a severe impact on patients’ quality of life and sociability [12,13,14]. The prevalence of SZ is approximately 1% of the population, and the heritability was calculated to be 79% [15]. As summarized in a recent review, several pharmacological, clinical, and genetic studies could correlate (i) glutamatergic dysfunction in SZ and NMDAR hypofunction in humans, (ii) the schizophrenia-like symptoms due to the autoimmune response against the extracellular domain of NMDARs, (iii) the exacerbation of SZ symptoms in patients administered with glutamatergic receptor antagonists, and (IV) *de novo* copy number variation (CNV) encoding NMDAR subunits and other proteins in the postsynaptic density (PSD) in patients with increased risk of SZ (for a review, see [15]). As summarized in that review, a meta-analytic study reported a significant elevation of glutamate + glutamine (Glx) in glutamatergic transmission in the limbic system, but no significant difference in glutathione—a tripeptide synthesized from glutamate, cysteine, and glycine in proton magnetic resonance spectroscopic imaging (1H-MRSI) studies. Brain imaging and EEG recordings supported the involvement of the glutamatergic system, NMDARs in particular, in SZ (for a review, see [15]). Several studies using postmortem brain tissues from SZ patients showed variable changes in mRNA and protein levels for iGluRs and mGluRs in different brain regions (for reviews, see [15,16,17]), and in 2012, a study of the Korean population identified *GRIA1* variants, the gene for the AMPAR subunit GluA1, as a SZ risk gene [18].

This strong correlation between genetically based impairments of the fast glutamatergic system and SZ led to several neurocircuitry hypotheses. One main experimentally based hypothesis is that the hypofunction of NMDARs in cortical fast-spiking parvalbumin interneurons leads to changes in cortical network oscillations [19,20,21], pointing to a communication impairment in the inhibitory and excitatory systems as an underlying mechanism for neuropsychiatric disorders; this was experimentally verified for NMDAR hypomorphic, NMDAR knockout mice [22] and conditional NMDAR mutant mice. In mice lacking the NMDAR, specifically in parvalbumin-expressing neurons, the hippocampal–prefrontal coherence was altered, indicating a disturbed excitatory-inhibitory balance [19,23,24]. In mice with genetic depleted CP+AMPARs, the SZ-like phenotype was consistently confirmed in several studies [25,26,27] and the SZ phenotype was correlated with the loss of NMDAR-dependent LTP in hippocampal CA1 cells in adults [11,28] (for a review of genetic SZ mouse models with glutamate receptor deficiencies, see [29]). 

In summary, data from clinical, pharmacological, and genetic studies strongly implicate the glutamatergic system, and in particular the Ca^2+^ signaling of NMDARs and CP+AMPARs, as the site of many of the abnormalities of brain processes that typically occur in SZ.

### 2.2. Autism Spectrum Disorder (ASD)

Another prominent neuropsychiatric disorder that is genetically inherited or caused by a *de novo* gene variant is ASD. Unlike SZ, ASD has a clear neurodevelopmental component. The symptoms typically appear before 3 years of age and are characterized by reduced social interactions, limited interest in communication, and repetitive patterns of behavior [30]. ASD is usually associated with other neuropsychiatric disorders, including, but not limited to, intellectual disability, anxiety, and attention-deficit hyperactivity disorder [31]. The heritability is between 70–90%, with a prevalence of around 1.5% in developed countries [32,33,34]. The high burden of ASD on society is further increased by the fact that its pathophysiology is largely unclear, and that effective therapies for the core symptoms of the disorder are not yet available [35].

The dysfunction of the glutamatergic system has been central to studies of neurotransmitter involvement in ASD. Both hyper- and hypo-glutamate models have been proposed on the basis of a variety of factors, such as ASD phenotypes, patient populations, experimental methods, and the brain regions studied [36,37,38]. The hyperglutamate theory was supported by the increased level of serum and plasma glutamate in children and adults with ASD [39,40,41,42,43,44,45,46] (for a review and meta-analysis, see [47,48]). Moreover, in the valproic acid (VPA)-induced ASD animal model, an upregulation of the GluN2A and GluN2B subunits of the NMDARs was observed with a corresponding activity-dependent long-term enhancement of synaptic transmission (LTP) [49], and an AMPAR antagonist restored the social behavior [50]. Several other studies confirmed the therapeutic effectiveness of iGluR antagonists, including topiramate (an antagonist of AMPARs/KARs) [51,52], memantine and amantadine (NMDAR antagonists) [53,54,55,56], and acamprosate (antagonist of NMDARs and mGluR5 [57,58]) in patients with ASD.

The alternative hypoglutamate theory was based on the dysfunction of the glutamate receptors in ASD and on the pharmacological effects of glutamatergic agonists in rescuing some ASD symptoms. Further evidence for a hypoglutamatergic state in ASD is provided by the therapeutic effects of piracetam, a positive AMPAR modulator [59]. Additionally, in several animal models of ASD, NMDAR signaling appears disrupted, mainly through a hypofunction mediated by the downregulation of the principal NMDAR subunit GluN1 (for a review, see [38]). Interestingly, the NMDAR agonists, e.g., D-cycloserine, which is known to modulate glutamatergic transmission [60], have been shown to improve sociability in patients with ASD [61,62,63] and in mouse models with an ASD-like phenotype [64,65,66,67,68]. In addition to the rescue of social behavior, D-cycloserine was also reported to be effective in attenuating stereotypic symptoms in adolescents and young adults with ASD [69]. AMPAR-positive allosteric modulators were able to rescue social impairment in *Cntnap2* knockout mice [50]. Various animal models of ASD have revealed alterations in the expression of glutamatergic receptors, along with their functions (for a review, see [38]).

In patients with fragile X syndrome (FXR), a leading genetic cause of autism, a hypofunction of synaptic AMPARs is postulated to be responsible for the intellectual disability (ID) and social–affective symptomatology of FXR patients. Numerous studies of *Fmr1* mouse models showed that AMPAR trafficking to the synapse is impaired in the absence of fragile X mental retardation 1 protein in *Fmr1* KO mice (for reviews, see [70,71]). 

Due to the low availability of postmortem human tissues, only a few postmortem studies investigated the expression of iGluRs and mGluRs in ASD patients. These studies revealed several alterations in the expression of glutamatergic receptors in multiple brain regions [72,73,74,75] (for a review, see [47]). Genetic studies clearly implicate glutamate receptors in ASD, including NMDARs [76,77,78,79], KARs [80,81,82], AMPARs [83,84], and mGluRs [85] (for a review, see [47]). Many genes expressing molecular components related to the glutamatergic system have been associated with ASD, including *NRXN1*, *2*, and *3* [86,87,88,89], *NLGN1*, *3*, and *4* [90,91,92,93], *CNTNAP2* [94,95] and *SHANK1*, *2*, and *3* [88,96,97,98,99,100] (for a review, see [101]). 

Concerning the glutamate level, several in vivo neuroimaging studies have revealed inconsistent alterations in the levels of glutamate and glutamine in various brain regions, including the cortex and basal ganglia of ASD patients [102,103,104,105,106,107,108,109,110]. For example, in vivo single-voxel and 1H-MRSI detected hyperglutamatergia (increased glutamatergic metabolites) in the pregenual anterior cingulate cortex in children and adolescents with ASD [102]. 1H-MRSI studies of non-clinical samples found that an increased glutamate/GABA^+^ ratio in the right hemisphere superior temporal region was correlated with a higher expression of the social disorganization, a shared phenotype within the autistic and schizotypal spectrum [111]. On the other hand, patients with ASD showed significantly lower glutamate concentration in the right anterior cingulate cortex (ACC) [103]. Additionally, translational 1H-MRSI showed a reduced glutamate concentration in the striatum of ASD patients, which was correlated with the severity of social dysfunction, implying that this endophenotype is clinically significant [35]. This reduction was also found in a VPA mouse model of ASD and mice and rats carrying *Nlgn3* mutations, but not in other ASD rodent models [35]. These translational data support the involvement of glutamatergic dysfunction in the corticostriatal pathway in the pathophysiology of ASD.

In summary, studies on humans and rodent models indicate that the glutamatergic system dysfunction via alterations in glutamatergic receptor expression, trafficking, and their synaptic/extrasynaptic localization leads to imbalanced excitatory transmission and alterations in both NMDAR-mediated synaptic development and plasticity and mGluR-mediated signal transduction. All these amendments appear to play a significant role in the pathophysiology of ASD [112].

## 3. Activity-Induced Modulation of Synaptic Ultrastructure

**Figure 1:**
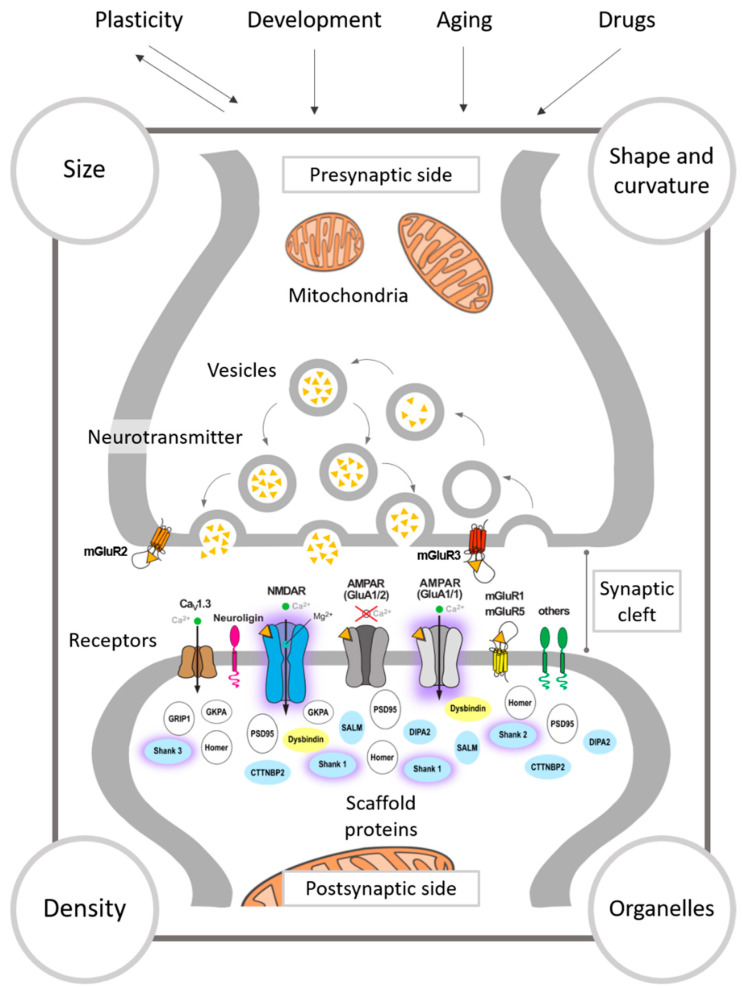
Factors affecting synaptic transmission. Physiological factors that affect synaptic transmission such as synaptic plasticity, development, and aging, and other non-physiological factors such as drug administration, have a synaptic ultrastructural correlate. Morphological changes include the density, size and shape of synapses, or affect organelles involved in synaptic transmision such as mitochondria. The proteins affected in the different animal models that are discussed in this re-view are colored according to the disorder, yellow for schizophrenia (see Table 1), and blue for autism spectrum disorder (see Table 2). Important key players in ASD and SZ are encircled in purple.

The study of neurotransmission has led to the conclusion that physiological properties can have a structural correlate, and that different morphological parameters modify the synaptic signal. The main physiological factors altering the structure of synaptic transmission are synaptic plasticity (for reviews, see [113,114]), development [115,116] (for reviews, see [117,118,119]), and aging [120,121], but other elements such as drug administration have also been shown to affect the synaptic structure [122] (Figure 1). The morphological changes can be directly related to synapses, such as modifications in their density and structure, or affect other organelles involved in synaptic transmission such as mitochondria and the endoplasmic reticulum. Here, we will focus on the changes happening at the level of synapses, briefly describing the changes occurring in the presynaptic side, and focusing on the postsynaptic modifications of the glutamatergic system as well as on the density of synapses. Later, we will describe the relevance of mitochondria in synaptic transmission.

### 3.1. The Presynapse

Regarding the presynaptic ultrastructure, it was shown in early studies on *Aplysia* that the presynaptic compartment of synapses could be remodeled, leading to an increase in the number, size, and vesicle complement of the active zones during long-term memory formation [147]. Later studies have revealed similar mechanisms in other species, such as *Drosophila*, where the readily releasable pool of vesicles can be dynamically modulated during plasticity [148,149], or in rodents, where the regulation of presynaptic scaffold proteins affects the availability of vesicles [150], and these can be replenished at different rates [151].

### 3.2. The Synaptic Cleft

It has been proposed, based on computer simulations, that changes in the width of the synaptic cleft alter synaptic function [152]. However, very little experimental research has been done to date on this matter. Glebov et al. (2016) showed a slight reduction in the width of the synaptic cleft after silencing neurons [153], and similar changes have been described in a disease model in which synaptic function is altered [132]. On the other hand, other models of synaptic malfunction exhibited opposite effects [131,139], and further research is needed.

### 3.3. The Postsynapse

Structural changes have been widely described in mammalian postsynapses, mainly in the glutamatergic system, in both the neocortex and hippocampus.

#### 3.3.1. Synaptic Size

The size of the synaptic junction has been described as a structural correlate of its function. The amplitude of synaptic currents correlates with the volume of dendritic spines [154,155]. In turn, dendritic spine volume strongly correlates with synaptic size [156]. Release probability also scales with synaptic size [157], as well as synapse stability over time, thus, larger synapses survive longer than smaller ones irrespective of synaptic activity [158]. It has been proposed that large synapses represent physical traces of long-term memory, while smaller synapses would be preferential sites for LTP induction [159,160], which has been also supported by simulation studies [160,161]. However, the size of synapses undergoes significant spontaneous changes [162], challenging the notion that they could be stable traces. Thus, new theories have arisen moving the focus of memory storage from individual synapses to network connections [163,164]. In fact, synapses are not distributed in two groups with clearly different sizes. Instead, the sizes of synaptic junctions follow a continuous distribution with a single peak and a long tail to the right, which fits a log-normal distribution both in the neocortex and hippocampus [165,166,167,168]. If, as commented above, different functions are performed by synapses of different sizes, there would be a continuous transition between the two types. It is interesting to note that other synaptic parameters, such as the amplitude of unitary excitatory postsynaptic potentials (EPSPs) [169,170] and spike transmission probability [171], also follow log-normal distributions (for a review, see [172]). Model experiments also suggest that larger synapses would not only evoke larger responses but would also be more homogeneous and reliable than smaller ones (i.e., their stochastic variability is reduced when compared to smaller synapses) [173,174].

In any case, the amplitude of postsynaptic response does not depend only on the size of the synapse but also the morphology of dendritic spines [175,176], as well as the geometry of postsynaptic dendrites [177,178]. Another important factor is the concentration of postsynaptic receptors. In the somatosensory cortex of rats, synapses of different sizes express a constant density of AMPARs, so the larger the surface of the PSD, the higher the absolute number of AMPARs [179]. In the hippocampus, however, different types of synapses have different AMPAR content, and the number of AMPARs scales with the synaptic size with different slopes [180]. The fact that NMDARs tend to be more concentrated in smaller synapses [179,181] increases the complexity of the relationship between synaptic size and function. Therefore, although it is clear that the synaptic size plays an important role in synaptic behavior, other factors, including dendritic spine volume, the geometry of the postsynaptic element, or the density of postsynaptic receptors, must also be considered.

#### 3.3.2. Shape of Synapses

Another morphological trace related to synaptic transmission is the shape of the synaptic junctions. Synapses can show a wide range of morphologies that have been classified into four main types: macular (when they are disk-shaped), perforated (with one or more holes in the PSD), horseshoe (when the perimeter is tortuous and horseshoe-shaped with an indentation), and fragmented (when the PSD is divided into two or more fragments) [182,183,184,185]. The different shapes also imply differences in the size of synapses: macular synapses have a smaller mean area than more complex morphologies, although their distributions greatly overlap [168].

The role that the shape of synapses plays in synaptic transmission is still under debate. Some findings support the notion that complex morphologies correspond to more active synapses; for example, it has been shown that in the hippocampus, there is an increase in the proportion of fragmented synapses after the induction of LTP [186,187]. It has also been shown that the shape of synapses might affect the proportion of postsynaptic receptors; for example, in the stratum radiatum of the hippocampal CA1 of adult rats, perforated synapses show a higher amount of AMPARs and NMDARs than nonperforated ones. This suggests that perforated synapses may evoke larger postsynaptic responses, and hence contribute to the enhancement of synaptic transmission associated with some forms of synaptic plasticity [188]. However, these differences may only correspond to the different sizes and might be independent of the shape of the synaptic junction.

#### 3.3.3. Curvature of the Synaptic Apposition Surfaces

In the early 1980s, synapses were classified, based on the curvature of their synaptic junction, as positive (when it was curved into the presynaptic terminal, in posterior studies referred to as convex), flat, and negative (when it was curved towards the postsynaptic side—later referred to as concave) [189]. This study described a shift towards more positive junctions as an indication of the increasing maturity of the synapses, but they also related the degree of curvature to the use of the synapse: junctions with pronounced negative curvatures would be non-functional, while flat synapses would be more active [189]. This theory was supported by the fact that the administration of barbiturates changed the curvature of synapses towards more curved shapes [190]. Later studies have shown that not only a variety of anesthetics [191] but also other parameters influence the curvature of synapses in healthy individuals, such as age [185,192] and nutrition [193], making it difficult to draw a conclusion about its relevance. The latest studies performed in rodent hippocampus suggested that changes in synaptic curvature may influence synaptic efficacy associated with long-term depression and LTP [194]. These changes could be due to the increased fusion of vesicles that would result in a growth of the postsynaptic membrane, or to changes in the cytoskeleton caused by the Ca^2+^ influx (for a detailed description of the possible mechanisms responsible for the changes in curvature, see [195]). Furthermore, another study showed that synaptic stimulation increased the curvature of the PSD, making it more convex [196], however, the characterization of synaptic curvature in this study (as in [189,190,193]) was performed in single sections instead of 3D reconstructions, which could be misleading, since the curvature of a single synapse can change from one section to another [195].

### 3.4. Synaptic Density

In the cortex, most synapses (90–98%) are established in the neuropil [197], which is composed of dendrites, axons, and glial processes. Of these, most are excitatory synapses mainly located on dendritic spines, while the rest correspond to inhibitory synapses, mainly established on the dendritic shafts [167,198,199].

The general view is that each spine establishes one synapse. However, some spines present multiple synapses [167,200,201,202,203], while others show a clear lack of them [204]. The presence of multiple synapses per dendritic spine [205,206,207], as well as a general increase in the total number of synapses [201], has been proposed to be linked to long-term memory formation. The fact that analyzing spines is much easier, due to their larger size, than studying synapses, and the correspondence between the volume of spines and excitatory synaptic strength have led to spines being considered a good structural criterion for excitatory synapses. Different studies have shown an increase in spine number in a variety of conditions, for example, in the mammalian cortex after the exposure to an enriched environment [208]; in the hippocampus after spatial training [209], running [210], and learning [211]; and in the brain of chicks after passive avoidance training [212]. Although an increase in spine density seems to be a trend after brain stimulation, its significance for activity must be considered carefully since, as stated above, changes in spines do not necessarily reflect changes in synapses.

### 3.5. Mitochondria

Mitochondria play an important role in synaptic transmission by providing most of the energy required by neurons and by acting as Ca^2+^ buffers [213,214,215,216]. The ATP provided by mitochondria at the synapse is used for maintaining the resting membrane potential, reversing the ion gradient after action and postsynaptic potentials, and for G-protein signaling, neurotransmitter recycling, and vesicle cycling [217]. Mitochondria are closely associated with synapses [218] and are transported along dendrites and axons to the regions of the cell that require a high energetic supply [219,220,221,222,223,224,225]. In the somatosensory cortex, the volume fraction of mitochondria located in axons and dendrites correlates with the local density of synapses, while the volume fraction of mitochondria located in non-synaptic processes does not [226]. The highest density of synapses is found in layer IV, a recipient of thalamic afferents [167]. Interestingly, layer IV also shows the highest density of mitochondria [226], which is associated with the synaptic vesicle pools to ensure the efficacy of the thalamic transmission [227]. This recruitment occurs through variations in the ADP/ATP ratio, but also other factors such as the activation of glutamate receptors [228] and changes in the concentration of Ca^2+^ affect mitochondrial mobility [214,215,222]. As Ca^2+^ buffers, mitochondria play a fundamental role in protecting the terminals by taking up cytosolic Ca^2+^ during repetitive stimulation [229,230]. Synapses and mitochondria are connected even further since there are proteins that regulate the mitochondrial distribution and also influence the formation and maintenance of spines and synapses, e.g., B-cell lymphoma extra large (Bcl-xL), which leads to more mitochondria at synapses and an increase in the synapse number and size when overexpressed [231] (for a review, see [216]).

## 4. Excitatory Synaptic Ultrastructure Alterations in Neuropsychiatric Disorders

The ultrastructural plastic organization of synapses affects the transmission, thus determining the mechanisms of adaptive or pathological behavior [124]. For example, as discussed before, the size of the active zone and the form of synaptic contacts correlate with the functional activity and maturity of the synapse. Therefore, the analysis of the synaptic ultrastructural alterations is important for the assessment of the functional state of synapses in different neuropsychiatric disorders.

### 4.1. Schizophrenia

Several studies identified synaptic ultrastructural alterations as evidence for glutamatergic dysfunctions in SZ patients (Table 1 and Figure 1) (for a recent review, see [232]). These alterations may depend on differences in the stage of illness, medication status, and brain regions. In the ACC, which plays a vital role in attention, executive functions and cognitive tasks [233], response conflict [234,235], error rate [236,237], and emotional processing [238], the total synaptic density was found to be decreased by 28% in individuals with SZ compared to healthy controls [123]. However, different types of synapses showed different alterations. The density of the glutamatergic axospinous synapses was reduced by 30%, while synapses on shafts showed no difference in SZ patients. The same study also revealed a selective decrease in the density of mitochondria in axon terminals forming excitatory axospinous synapses, specifically in layer III of the ACC [123], suggesting a decrease in cortical synaptic efficiency which can impact the cognitive functions controlled by the ACC activity.

In layer II of the anterior limbic cortex, a 128% increase in the density of total axospinous synapses and a decrease of 40% of synapses on the dendritic shaft were identified in SZ subjects without determining whether the decreased numbers are in excitatory or inhibitory synapses. These results suggest a disruption in the formation of connections in the neuronal assemblages of the anterior limbic cortex or a defective elimination of synapses during early adolescence, when synaptic pruning occurs by discarding weak and redundant synaptic connections, and strengthening the remaining synapses [239,240]. Regarding the structure of synapses, the same study revealed an increase of 14% in the convex synapses in SZ cases with a decrease in flat and concave synapses by 11% and 3%, respectively [124].

In the hippocampus, two electron microscopy (EM) studies morphometric studies reported a decreased number of glutamatergic axospinous synapses between the mossy fiber axon terminals and branched dendritic spines of pyramidal neurons in the CA3 region in SZ subjects. These results reflect a decrease in the efficacy of mossy fiber synapses in the CA3 hippocampal region, which can impair cognitive maintenance [125,126].

In the caudate nucleus and putamen, as parts of the dorsal striatum that process motor, cognitive, and limbic functions, a selective increase in the glutamatergic synaptic density was reported in SZ patients, suggesting an enhanced cortical excitatory input, as the striatum receives mainly excitatory inputs from the cortex and shows major GABAergic output [127]. The caudate nucleus and putamen are composed of patches embedded in a larger matrix [241]. The matrix compartment preferentially receives inputs from the motor and somatosensory cortices [242], as well as from the dorsolateral prefrontal cortex [243] which processes cognition, including working memory [244]. On the other hand, the patch compartment receives input from the limbic system [128].

The glutamatergic axospinous synaptic density was elevated in both the caudate matrix and the putamen patches in SZ cases on typical antipsychotic drugs [128]. However, the increase in total and excitatory synapses in the patch (but not the matrix) was confined to the treatment-resistant group, while the treatment respondent group had normal levels of synapses except for an increase in the density of glutamatergic axodendritic synapses in both the patch and matrix [129]. There is therefore evidence of altered glutamatergic activity in SZ with an association between striatal function, structure, and treatment response.

The nucleus accumbens acts as a central hub for integrating signals from several regions associated with SZ, including the prefrontal cortex, hippocampus, amygdala, and thalamus [245]. It can be divided into the core and shell. In SZ patients, the core showed an increased density of excitatory axospinous synapses, although they have smaller PSDs. In contrast, the shell did not present any difference from healthy brains. Similarly, mitochondrial density in the nucleus accumbens did not show any alterations in SZ. The frequency of large elaborate multi-perforated synapses was equally found in axospinous synapses and synapses on dendritic shafts and was not altered in SZ [130]. These large synapses play a role in the complex interconnectivity of the nucleus accumbens, which makes it a unique feature of the human brain [130,232].

Despite the presence of genetic rodent models with iGluR or mGluR depletions or mutations described as having an SZ-like phenotype, these studies mainly focused on the electrophysiological alterations (for a review, see [246]) with no synaptic ultrastructural details available except for one pharmacological and one genetic-induced rodent model of SZ. In a ketamine-induced SZ rat model, a reduction in the thickness and curvature of the synaptic interface and an increase in the synaptic cleft width in the posterior cingulate cortex were found, which could be rescued by the administration of vinpocetine, a nootropic phosphodiesterase-1 inhibitor [131]. In the sandy mouse line that harbors a spontaneously occurring deletion in the *Dtnbp1* gene and expresses no dysbindin protein, although the overall appearance of presynaptic terminals and spines in the hippocampal CA1 glutamatergic synapses was normal, quantitative analysis revealed a shift of the vesicle distribution to a ~10% larger size, with a normal count of docked vesicles, a reduced count of reserve pool vesicles, and a decreased width of the synaptic cleft [132]. The glutamatergic synapses in sandy mice also exhibited an increase in the thickness of PSDs.

The inconsistent synaptic ultrastructural alterations between human patients and rodent models as well as between the different rodent models of SZ point to the severe heterogeneity of this complex disorder. Moreover, it may indicate the limitation of the rodents to modulate such complex disorders. Indeed, on the behavioral level, some of the symptoms of SZ, such as auditory hallucinations and delusions, have not yet been modeled due to the difficulty of finding a correlate in animals. In contrast, deficits in sensory processing have proven more amenable to modeling in rodents, including sensorimotor gating. Therefore, face validity is vital to signify that a rodent model can recapitulate important anatomical, biochemical, neuropathological, or behavioral features of a neuropsychiatric disorder. Additionally, before generating a new rodent model, it is important to achieve a construct validation by recreating the same mutation in the gene that was found in the patient to dissect the heterogeneous state of these disorders.

In summary, human postmortem and rodent studies have revealed several glutamatergic synaptic ultrastructural alterations related to SZ, confirming the notion of synaptic involvement in SZ (for a summary of synaptic ultrastructural alterations in SZ, see Table 1 and Figure 1).

### 4.2. Autism Spectrum Disorder

In ASD, efforts to carry out comparative postmortem brain studies in patients were hindered by poor tissue preservation and small sample sizes. To our knowledge, only one EM study investigating the brains of ASD patients was performed, with a small sample size of adult brains from five ASD patients and four controls. This study did not investigate the synaptic structure but rather focused on the connectivity of myelinated axons in the prefrontal cortex, revealing significantly fewer large axons in the deep white matter below the ACC and much smaller axons in the superficial white matter of the same region, suggesting increased local connectivity and decreased long-range connectivity [133].

Data on synaptic ultrastructure changes are available only for several pharmacological and genetic-induced rodent models of ASD, which document the heterogeneity of ASD (Table 2 and Figure 1). The synaptic ultrastructural alterations in two examples of the pharmacologically induced rodent models of ASD will be described in detail. An ASD rat model of prenatal exposure to VPA exhibited a blurred and thickened structure of the synaptic cleft without clearly marked pre-and postsynaptic membranes. Synaptic vesicles were found to be greatly reduced or completely absent in the presynaptic terminals in the cerebral cortex and hippocampus, with altered mitochondrial shapes and fused mitochondrial cristae and membrane [134]. In a propionic acid mouse model of ASD, multiple synaptic alterations were observed in the CA1 region of the hippocampus, including the presence of some atypically enlarged presynaptic terminals with a reduced density of synaptic vesicles and short active zones [135].

For the genetic-associated ASD rodent models, studying a mouse model with a human 15q11-13 chromosomal duplication revealed that the size of the PSD, the spine head volume, and spine head and spine neck widths were reduced in layer II/III of the somatosensory cortex, indicating a poor maturation of spines. The reported tendencies towards the increased density of spine synapses, reduced densities of shaft synapses with a trend towards increased fractions of filopodia, thin spines, and reduced fractions of mushroom spines support the idea of an altered balance between excitatory and inhibitory synapses in this model [136].

The SHANK protein family comprises a group of postsynaptic scaffolding proteins that play a vital role in the formation, organization, and signaling of glutamatergic synapses [64], acting as the central organizer of the postsynaptic proteins. Since mutations of all three members of the *SHANK* gene family are associated with ASD, the ultrastructural alteration in *Shank* mutant mice was expected to give information on the functional impairment of the network and to provide a link to the alterations found in SZ, in particular *SHANK2* variants associated with SZ [247]. The deletion of *Shank1* in mice displaying an ASD-like phenotype leads to smaller spines, thinner PSDs, and a weak synaptic transmission in the hippocampal CA1 region [137]. In *Shank3B^−/−^* mice revealing an ASD-like phenotype, the thickness and length of the PSD in the striatum were decreased, with a reduction in the spine density accompanied by a reduced excitatory synaptic transmission, pointing to the disruption of glutamatergic signaling [138]. In another ultrastructural study in the hippocampal CA1 field in *Shank3*-deficient mice, the presence of synaptic pathology at different developmental stages (5 weeks and 3 months of age) was assessed. *Shank3^+/–^* heterozygote mice had significantly more perforated synapses at 5 weeks than at 3 months of age and significantly more than 5-week-old controls, indicating that the ultrastructural morphological alterations affecting synaptic structure may occur in an age-dependent manner in *Shank3*-deficient mice [248].

LRFN2/SALM1 is a PSD-95-interacting synapse adhesion molecule [249]. In the *Lrfn2*-deficient mouse model of ASD, the CA1 stratum radiatum of the dorsal hippocampus revealed an increased ratio of perforated synapses to total excitatory synapses. Moreover, the synapses had a shorter PSD length but a similar thickness and a wider synaptic cleft. Furthermore, an unstable spine structure was suggested by the presence of oddly shaped, spinule-like spines and perforated PSDs [139].

The ASD-associated cortactin binding protein 2 (CTTNBP2) is known to regulate the subcellular distribution of synaptic proteins, such as cortactin, thereby controlling dendritic spine formation and maintenance [250]. In the *Cttnbp2^−/−^* mouse model of ASD, in all regions of the dorsal hippocampus, these mice showed a reduction in the length and thickness of PSDs, the count of presynaptic vesicles, and the ratio of vesicle number to PSD length [140].

The ASD candidate gene *DIP2A* (disconnected-interacting protein homolog 2 A) encodes for a protein that is localized to dendritic spines in excitatory neurons [141]. The deletion of *Dip2a* in mice exhibited a postsynaptic structure with a stubby appearance and flattened PSD compared to an enlarged postsynaptic terminal with a distinct neck and abundant PSDs in control mice [141]. These results were accompanied by a defect in spine morphology and synaptic transmission, which may be mediated by PSD size and glutamate receptor dysfunction.

Calsyntenin-2 (CLSTN2) is a synaptic protein that belongs to the superfamily of cadherins and has an important function in learning and memory [251]. In the *Clstn2^−/−^* mouse model of ASD, the synaptic ultrastructural analysis revealed a reduction in the density of inhibitory synapses both in the medial prefrontal cortex (MPFC) and the hippocampus, as well as a general increase in negatively curved PSDs. The MPFC also showed a reduction in the length of perforated synapses as well as in the width of the synaptic cleft PSD, presenting a surprisingly increased number of synaptic vesicles. In contrast, the presynaptic area of the hippocampal neurons was not changed in *Clstn2* knockout mice, but the number of synaptic vesicles was significantly reduced [142].

In addition to the aforementioned synaptic proteins, nuclear proteins associated with ASD can also affect the synaptic ultrastructure, e.g., vaccinia-related kinases (VRKs) that play a major role in cell signaling, cell cycle progression, apoptosis, and neuronal development. In the hippocampal CA1 region of a VRK3-deficient mouse model of ASD, prominent reductions in PSD length and thickness were revealed [143].

Adding another layer of complexity, a mouse model of Phelan–McDermid syndrome causing autistic phenotypes with a deficiency of mitogen-activated protein kinase 8 interacting protein 2 (MAPK8IP2/IB2), which plays an important role in regulating the ratio of AMPARs to NMDARs at glutamate synapses, revealed no synaptic ultrastructure alterations of cerebellar glutamatergic synapses and featured normal synaptic clefts and postsynaptic densities and abundant presynaptic vesicles [252]. However, despite the normal ultrastructure, a larger NMDAR-mediated current and enhanced intrinsic excitability and LTP were revealed in this mouse model [253].

Although altered dendritic spine morphology is a hallmark of FXS, the degree of spine abnormalities observed in the *Fmr1* KO mouse model is variable (for a review reporting the spine phenotypes in different brain regions of *Fmr1* KO, see [144]). Additionally, in more recent studies, a significant increase in the diameter of secondary dendrites, an increase in dendritic spine density, a decrease in mature dendritic spines and less mature postsynaptic densities were revealed in the hippocampal CA1 region of adult *Fmr1*^−/−^ mice [145]. In the primary motor cortex of *Fmr1^−/−^* mice, a normal density but higher turnover rate of dendritic spines were shown [146].

In summary, several pieces of evidence from rodent studies have revealed heterogeneous synaptic ultrastructural alterations related to ASD, supporting the hypothesis that ASD is, in part, the consequence of a developmental synaptopathy (for a summary of synaptic ultrastructural alterations in ASD, see Table 2 and Figure 1). Improving the quality of postmortem tissue preparation from ASD patients by the development of advanced methods can confirm the synaptic ultrastructural changes revealed in rodent models of ASD and pave the way for dissecting the heterogeneity of the disorder. Moreover, it will facilitate finding ultrastructural alterations that are common with other neuropsychiatric disorders and contribute to their pathophysiology.

## 5. Conclusions

Numerous experimental and EM-imaging methods have shown that in genetically and pharmacologically caused SZ and ASD, glutamate concentration, glutamatergic receptor expression, and the ultrastructure of glutamatergic synapses are differentially affected in different brain regions, leading to alterations in synaptic signal transmission. Various molecular, physiological, and structural changes of synapses have already been shown to occur during brain development or learning. Those studies identified the glutamatergic neurotransmission as an essential mechanism for an activity-dependent modulation of synapses and the formation (learning) or stabilization (memory) of neural networks. For those network changes, the Ca^2+^ influx at postsynaptic NMDARs and AMPARs and the release of intracellular Ca^2+^ at the activated synapse are essential to initiate the required modification of the synapses that experienced activation through depolarization of the postsynaptic membrane. If the intracellular Ca^2+^ level rises too far, neuronal death will result [254]. Thus, the amount and type of Ca^2+^ increase after glutamate stimulation determines whether and which molecular changes will be activated in the neuron. This could be associated with structural changes in the synapse(s) and increased/decreased excitability. In SZ and ASD, the glutamatergic signal transduction in neuronal networks that controls social and other behaviors is dysregulated. These structural alterations can have a clear impact on synaptic neurotransmission and plasticity, which lead to neuronal circuit defects. The maintenance of cognition and normal behaviors is heavily dependent on the precise formation of mature neuronal circuits. For example, the increase in glutamatergic synaptic density can cause hyper-excitability of cortical circuits consistent with multiple lines of evidence that implicate imbalances in excitatory and inhibitory activity as a shared pathophysiological mechanism in SZ and ASD. Additionally, as synaptic size and shape can have an effect on the number of AMPARs and NMDARs, key components of learning and memory, the alteration of these factors can explain the cognition impairment and memory dysfunction in these neuropsychiatric disorders. However, to understand the different features of both neuropsychiatric disorders and their individual heterogeneity, a more detailed picture of the neuronal network and more comparative ultrastructural information are required. Importantly, structural and functional studies on SZ and ASD also show that the glutamatergic synapses do not appear to lose their plasticity during maturation and learning. In rodent models and human patients, the impaired glutamatergic synaptic plasticity and social behavior can be at least partially normalized genetically or by compensatory medication, as indicated by the rescue of the social impairment via restoration of Shank3 expression in adult *Shank3* KO mice [255] and by the treatment of adult *Shank2* KO mice with an NMDAR agonist, D-cycloserine [65]. Additionally, both experimental and medical interventions were associated with normalized social behavior but also improved learning abilities in adult animals and patients. Detailed synaptic ultrastructural analysis of AMPAR and NMDAR KO mice are necessary to demonstrate that changes in synaptic structures are associated with SZ and ASD phenotypes recognized in AMPAR and NMDAR KO mice, as noticed in patients and the other mouse models for ASD and SZ; in particular, the loss of NMDAR in hippocampal CA3 to CA1 synapses and GluA1 receptor plasticity is associated with impairments in decision making, which depends on the recognition and correct evaluation of environmental stimuli but is not involved in the long-term memory formation [256,257].

Since astrocytes in tripartite synapses also influence synaptic transmission and react to inflowing and intracellularly released Ca^2+^ signals, the role of astrocytes and their mitochondria in neuropsychiatric diseases should be investigated.

## Figures and Tables

**Table 1:** Synaptic ultrastructural alterations in schizophrenia. Different studies have assessed the synaptic ultrastructural changes of synaptic transmission in schizophrenia in postmortem human tissues and rodent models (either chemical or genetic). The affected proteins are shown in Figure 1. ↑stands for increased, ↓stands for decreased. CA1: Cornu ammonis area 1, CA3: Cornu ammonis area 3, PSD: postsynaptic density.

Schizophrenia	
**Humans**	
**Brain Region**	**Ultrastructure Modifications**
Anterior cingulate cortex	↓ density of axospinous synapses and axonal mitochondria [123].
Anterior limbic cortex	↑ density of axospinous and convex synapses; ↓ density of synapses on shafts, flat and concave synapses [124].
Hippocampus CA3	↓ density of axospinous synapses [125,126].
Caudate and putamen	↑ density of in axospinous synapses the caudate matrix and putamen patches [127,128,129].
Nucleus accumbens	↑ density and ↓ size of axospinous synapses in the core [130].
**Rodents**	
**Rodent Models**	**Ultrastructure Modifications**
Ketamine	In posterior cingulate cortex, ↑ thickness and curvature of the synaptic interface; ↑ synaptic cleft width [131].
*Dtnbp1^−/−^*	In CA1, ↑ vesicle size and thickness of PSD; ↓ vesicles of reserve pool and width of synaptic cleft [132].

**Table 2:** Synaptic ultrastructural alterations in autism spectrum disorder. Different studies have assessed the synaptic ultrastructural changes of synaptic transmission in autism spectrum disorder. Only one has been done in postmortem human tissue. Rodent models are either chemical or genetic. The affected proteins are shown in Figure 1. ↑stands for increased, ↓stands for decreased. Full names of brain regions, genes, proteins, and drugs are as in the main text and abbreviation list. Cx: cortex, HC: hippocampus, MPFC: medial prefrontal cortex, CA1: Cornu ammonis area 1, PSD: postsynaptic density.

Autism Spectrum Disorder	
**Humans**	
**Brain Region**	**Ultrastructure Modifications**
Anterior cingulate cortex	↑ large axons in deep white matter, ↑ small axons in superficial white matter [133].
**Rodents**	
**Rodent Model**	**Ultrastructure Modifications**
VPA	In Cx and HC, blurred and thickened synaptic cleft; ↓ synaptic vesicles; altered mitochondrial morphology [134].
Propionic acid	In CA1, few atypically enlarged presynaptic terminals; ↓ density of synaptic vesicles and short active zone [135].
15q11-13 duplication	In somatosensory Cx, ↓size of PSD, spine head volume ↓width, spine neck width ↓density of shaft synapses and mushroom spines; ↑ density of axospinous synapses and filopodial spines [136].
*Shank1^−/−^*	In CA1, ↓smaller spines and thinner PSD [137].
*Shank3B^−/−^*	In striatum, ↓ thickness and length of PSD and spine density [138].
*Lrfn2^−/−^*	In CA1, ↑perforated synapses and synaptic cleft width; ↓PSD length; oddly shaped and spinule-like spines [139].
*Cttnbp2^−/−^*	In dorsal HC, ↓PSD length and thickness and synaptic vesicle count [140].
*Dip2a^−/−^*	In Cx, a stubby postsynaptic structure and flattened PSD; defect in spine morphology [141].
*Clstn2^−/−^*	In MPFC, ↑ density of inhibitory synapses, ↑ negative curved PSD. In ADD, ↑ size of perforated PSD; ↑ density of synaptic vesicles. In HC, ↓ density of synaptic vesicles [142].
*Vrk3^−/−^*	In CA1, ↓ PSD length and thickness [143].
*Fmr1^−/−^*	For a review reporting the spine phenotypes in different brain regions, see [144]. In CA1, ↑ diameter of secondary dendrites and dendritic spine density; ↑ mature dendritic spines and ↑ mature postsynaptic densities [145]. In the primary motor Cx, normal density but ↑ turnover rate of dendritic spines [146].

## Data Availability

Not applicable.

## Abbreviations

**1-H-MRSI**, Proton magnetic resonance spectroscopic imaging; **ACC**, Anterior cingulate cortex; **AMPAR**, α-amino-3-hydroxy-5-methylisoxazole-4-propionate receptor; **ASD**, Autism spectrum disorder; **Bcl-xL**, B-cell lymphoma extra large; **CA1**, Cornu ammonis area 1; **CA2**, Cornu ammonis area 2; **CA3**, Cornu ammonis area 3; ***Clstn2***, Mouse gene for calsyntenin-2; **CNV**, Copy number variation; ***CNTNAP2***, Human gene for contactin-associated protein-like 2; **CP-AMPAR**, Ca^2+^-impermeable AMPA receptors; **CP+AMPAR**, Ca^2+^-permeable AMPA receptors; **CTTNBP2**, Cortactin-binding protein 2; **Cx**, Cortex; **DIP2A**, Disconnected-interacting protein homolog 2A; ***DIP2A*** Human gene for disconnected-interacting protein homolog 2 A; **EPSP**, Excitatory postsynaptic potential; **FXR**, Fragile X syndrome; **GluA1**, Glutamate receptor GluA1 receptor subunit 1; **GluN1**, Glutamate receptor NMDA receptor subunit 1; **GluN2A**,**B**,**C**,**D**, Glutamate receptor NMDA receptor subunit 2A,B,C,D; **GluA1**,**2**,**3**,**4**, Glutamate receptor AMPA receptor subunit A,B,C,D; ***Gria1***, Mouse gene for GluA1; ***Grin1***, Mouse gene for GluN1; **iGluR**, Ionotropic glutamate-gated receptor; **ID**, Intellectual disability; **HC**, Hippocampus; **KAR−** Kainate receptor; ***LRFN2/SALM1***, Synaptic adhesion-like molecule/leucine-rich repeat and fibronectin type III domain containing; **LTP**, Long-term potentiation; **mGluR**, Metabotropic glutamate receptor; **MMRRC**, Mutant Mouse Resource and Research Centers by NIH; **MPFC**, Medial prefrontal cortex; ***NLGN***, Human gene for neurolegin; ***NMDAR***, *N*-methyl-d-aspartate receptor; ***NRXN***, Human gene for neurexin; **P12**, Postnatal day 12; **PSD**, Postsynaptic density; **SH3**, Src-homology domain; **SHANKs**, SH3 and multiple ankyrin domain proteins; ***SHANK1***, ***2***, ***3***, Human SHANK1, 2, and 3 genes; ***Shank1***, ***2***, ***3***, Mouse genes for SHANK1, 2, and 3 proteins; **SZ**, Schizophrenia; **VPA**, Valproic acid; **VRK**, Vaccinia-related kinase.

## Mouse lines

***Clstn2^−/−^***, global depletion Clstn2—not listed—a CRISPR-Cas9-edited KO mouse line is available at MMRRC: Clstn2^em1(IMPC)J^ (MGI:5766210);

***Cttnbp2^−/−^***, global depletion of Cttnbp2—not listed—a CRISPR-Cas9-edited KO mouse line is available: Cttnbp2^tm1a(KOMP)Wtsi^ (MGI:4455588);

***Dip2a^−/−^***, global depletion of Dip3a—not listed—a CRISPR-Cas9-edited KO mouse line is available: Dip2a^em#Ywz^ (MGI:5787957);

**Dtnbp1^−/−^**, DtnbP1 (floxed mice B6(Cg)-Dtnbp1tm1c(EUCOMM)Hmgu/DtlJ are available at Jackson labs Stock 030840);

***Fmr^−/−^***, global Fmr depletion: Fmr1^tm1Cgr^ (MGI:1857169) or Fmr1^tm2Cgr^ (MGI:245108);

***Gria1^−/−^***, global GluA1 depletion: Gria1^tm1Rsp^ (MGI:2178057);

***Grin1^neo/neo^***, hypomorophic GluN1 expression: Grin1^tm1Bhk^ (MGI:1928280);

***Grin1^f/f^/PV-Cre^−/−^***, GluN1 depletion in parvalbumin-positive neurons: Grin1^tm1Rsp^ (MGI:3611337)/Pvalb^tm1(cre)Arbr^ (MGI:3590684);

***IB2^−/−^***, global depletion of Mapk81P/Ib2: Mapk8ip2^tm1.1Gol^ (MGI:4867721);

***Lrfn2^−/−^***, global depletion of Lrfn2/Salami—not listed—a similar 2lox mouse strain is available from Jackson labs: B6J(Cg)-Lrfn2^tm1.2Csbd^/Mmjax (MMRRC Stock No: 50502-JAX);

***Shank1^−/−^***, global Shank1 depletion: Shank1^tm1Shng^ (MGI:3762757); ***Shank3B^−/−^***, global depletion of the Shank3B isoform: Shank3^tm2Gfng^ (MGI:4949738);

***Vrk3^−/−^***, global depletion of Vrk3—not listed—a CRISPR-Cas9-edited KO mouse line is available at MMRRC: VRK3em1^(IMPC)MBP/MmUCD^ (MGI: 63152674).
